# Surgery-induced monocytic myeloid-derived suppressor cells expand regulatory T cells in lung cancer

**DOI:** 10.18632/oncotarget.14991

**Published:** 2017-02-04

**Authors:** Jun Wang, Liu Yang, Lu Yu, Yi-Yin Wang, Rui Chen, Jing Qian, Zhi-Peng Hong, Xiao-San Su

**Affiliations:** ^1^ Department of Anesthesiology, The First Affiliated Hospital of Kunming Medical University, Kunming, China; ^2^ Biomedical Research Center, The Affiliated Calmette Hospital of Kunming Medical University, Kunming, China; ^3^ Department of Pathology, The Affiliated Calmette Hospital of Kunming Medical University, Kunming, China; ^4^ Department of Laboratory Medicine, The Affiliated Calmette Hospital of Kunming Medical University, Kunming, China; ^5^ Department of Thoracic Surgery, The First Affiliated Hospital of Kunming Medical University, Kunming, China

**Keywords:** lung cancer, myeloid-derived suppressor cells, regulatory T cells, perioperative period, metastasis

## Abstract

While monocytic myeloid-derived suppressor cells (M-MDSCs) have been reported to induce the development of regulatory T cells (Treg), little is known about their correlation with Treg during perioperative period. Here, we demonstrated that the M-MDSCs expressing CD11b^+^CD33^+^HLA-DR^–^CD14^+^ in lung cancer patients after thoractomy significantly increased in comparison with preoperation, and their accumulation linearly correlated with an increase in Treg. Surgery-induced M-MDSCs, in addition to have high arginase activity, were more efficient in suppressing T-cell proliferation. Furthermore, the surgery-induced Treg expressed high levels of Foxp3, PD-1 and CTLA-4. Surgery-induced M-MDSCs were more potent in expending Treg when cocultured with autologous T cells *in vitro*. Using a lung metastasis mouse model, we demonstrated that the M-MDSCs at postoperative period were significantly increased and linearly correlated with Treg. We also showed that all-trans retinoic acid significantly inhibited the induction and proliferation of M-MDSCs, suppressed expansion of Treg, and finally prevented tumor metastasis in the mice after tumor resection. Receiver operating characteristic analyses revealed the superiority of surgery-induced M-MDSCs and Treg to those at preoperative period as a prognostic marker for lung cancer patients. Taken together, our results link the presence of surgery-induced M-MDSCs with the emergence of Treg and identify M-MDSCs and Treg derived postoperatively as potential indicators of tumor metastasis.

## INTRODUCTION

Myeloid-derived suppressor cells (MDSCs) are a heterogeneous population of myeloid progenitors including monocytes, granulocytes, and other cells that express both Gr-1 and CD11b in mice and suppress immune responses [1]. In human, MDSCs are characterized by the cell surface expression of CD11b, CD33, and low expression of HLA-DR [2]. The expression of CD14 has been used to characterize two major populations of MDSCs: monocytic MDSCs (M-MDSCs) expressing CD14^+^, and granulocytic MDSCs (G-MDSCs) expressing CD14^–^ [3, 4]. Studies in recent years have revealed that MDSCs can inhibit antitumor immunity by inducing regulatory T cells (Treg) and transforming growth factor (TGF)-β secretion to mediate T cell suppression [5–7]. Although the ability of surgery-induced MDSCs to promote angiogenisis and tumor growth has been described [8], it has not been established whether a possible interaction of surgery-induced MDSCs and Treg development exists after tumor resection. We therefore hypothesize that surgery-induced accumulation of MDSCs, in addition to induce T cell dysfunction [9], can expand Treg to further establish immunosuppression and promote tumor metastasis.

In an attempt to investigate the immune regulatory function of MDSCs during perioperative period, we detected the amount of peripheral G-MDSCs, M-MDSCs and Treg in lung cancer patients, and evaluated their inhibitory properties. Our results demonstrated that M-MDSCs increased after lung cancer surgery and surgery-induced M-MDSCs correlated significantly with gradually elevated numbers of Treg in circulation. These surgery-induced M-MDSCs were more efficient in expanding Treg than preoperative M-MDSCs *in vitro*. We also demonstrated that *all-trans* retinoic acid (ATRA) significantly inhibited the induction and proliferation of M-MDSCs, suppressed Treg generation, and finally prevented lung metastasis formation in the mice underwent tumor resection. Taken together, these data suggest that M-MDSCs mediate the expansion of Treg in lung cancer patients following tumor resection.

## RESULTS

### Positive correlation between M-MDSCs and Treg in lung cancer patients

**Figure 1:**
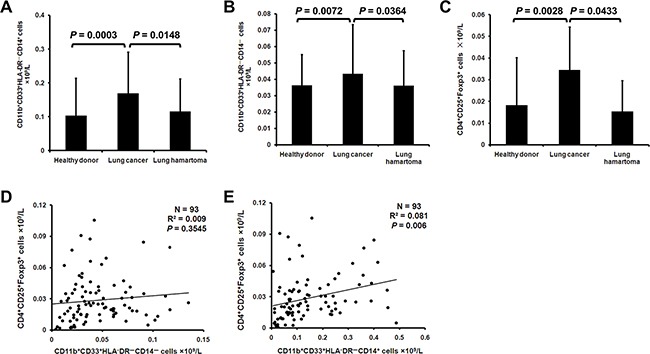
MDSCs and Treg are increased in lung cancer patients PBMCs from 41 healthy donors, 111 lung cancer patients and 18 lung hamartoma patients were analyzed for G-MDSCs, M-MDSCs and Treg. The concentration of CD11b^+^CD33^+^HLA-DR^–^CD14^+^ **A**. CD11b^+^CD33^+^HLA-DR^–^CD14^–^ **B**. and CD4^+^CD25^+^Foxp3^+^ **C**. cells in PMBCs was calculated. Correlation between the concentration of G-MDSCs **D**. M-MDSCs **E**. and Treg in lung cancer patients was analyzed.

When the MDSCs in peripheral blood mononuclear cells (PBMCs) of lung cancer patients were analyzed for CD14 expression, we noticed a significant increase in the concentration of CD11b^+^CD33^+^HLA-DR^–^CD14^+^ (M-MDSCs) and CD11b^+^CD33^+^HLA-DR^–^CD14^–^ (G-MDSCs) compared with healthy donors and lung hamartoma patients (Figure 1A, 1B). Meanwhile, lung cancer patients had a higher concentration of CD4^+^CD25^+^Foxp3^+^ cells (Treg) in PBMCs (Figure 1C). While there was no significant linear association between the G-MDSCs and Treg (Figure 1D), there was a significant association between the M-MDSCs and Treg (Figure 1E).

### Positive correlation between surgery-induced M-MDSCs and Treg in lung cancer patients

**Figure 2:**
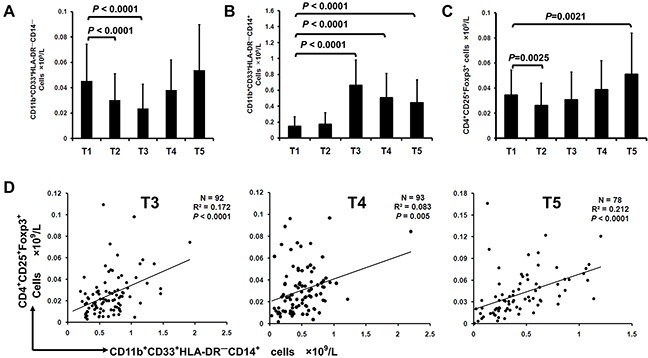
Surgery-induced M-MDSCs from lung cancer patients mediate expansion of Foxp3^+^ Treg *in vivo* Flow cytometric analysis of G-MDSCs, M-MDSCs and Treg in PBMCs of 111 lung cancer patients was performed at the time of preoperatoin (T1), intraoperation (T2), 1, 3 and 7 days (T3, T4 and T5) after tumor resection. Concentration of G-MDSCs **A**. M-MDSCs **B**. and Treg **C**. in PBMCs of lung cancer patients during perioperative period was calculated. **D**. Correlation between the concentration of M-MDSCs and Treg in lung cancer patients from T3 to T5 time point was analyzed.

We next evaluated the relationship between peripheral MDSCs and Treg at preoperation, intraoperation and on postoperative day 1, 3 and 7 (depicted as T1, T2, T3, T4 and T5, respectively) in lung cancer patients. While G-MDSCs decreased at T2 and T3, the concentration of M-MDSCs increased significantly at T3, T4 and T5 compared with T1 (Figure 2A, 2B). In parallel, the concentration of Treg was significant decreased at T2 and increased at T5 compared with T1 (Figure 2C). There was a significant linear association between the concentration of M-MDSCs and Treg at T3, T4 and T5 in lung cancer patients (Figure 2D). No correlation was found between G-MDSCs and Treg (data not shown).

### Immunosuppressive property of surgery-induced M-MDSCs and Treg in lung cancer patients

**Figure 3:**
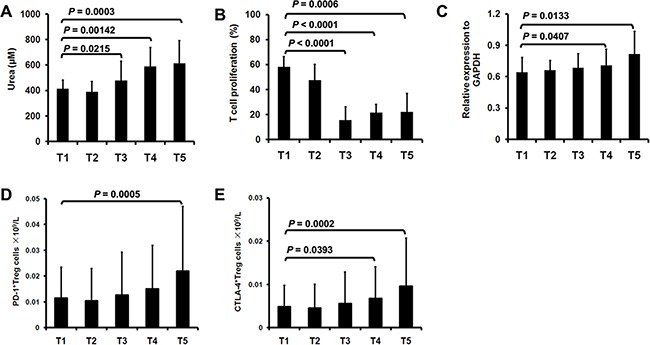
Immunosuppressive property of surgery-induced M-MDSCs and Treg **A**. M-MDSCs were sorted from lung cancer patients (n=16) during perioperative period and arginase activity was detected. **B**. CD3^+^ cells derived from lung cancer patients (n=16) were stimulated with anti-CD3 mAb in the presence of M-MDSCs. **C**. The Treg were sorted from lung cancer patients (n=16) and used for total RNA isolation and the expression of Foxp3 was assessed by quantitative real-time PCR. The concentration of PD-1^+^ **D**. and CTLA-4^+^ cells **E**. in Treg was analyzed from lung cancer patients (n=16) during perioperative period.

The suppressive activity of MDSCs has been associated with the metabolism of arginine to urea and ornithine [5]. We therefore detected arginase activity in surgery-induced M-MDSCs in lung cancer patients. As showed in Figure 3A, M-MDSCs isolated from T3, T4 and T5 had significant increased arginase activity compared with T1. We next evaluated the capacity of M-MDSCs to suppress T cell proliferation. In contrast with M-MDSCs isolated from T1, postoperative-isolated M-MDSCs demonstrated higher levels of T cell suppression (Figure 3B). These results suggest that surgery-induced M-MDSCs from lung cancer patients possess more potent immunosuppressive activity.

As a hallmark characteristic, the expression of Foxp3 in Treg was detected in lung cancer patients during perioperative period. Quantitative real-time polymerase chain reaction (PCR) analysis showed that the relative expression of Foxp3 in Treg was significantly increased at T4 and T5 compared with T1 (Figure 3C). Moreover, we stained immunoregulatory receptors programmed death-1 (PD-1) and cytotoxic T lymphocyte antigen-4 (CTLA-4) expressed on Treg. In line with the increase in the concentration of Treg, PD-1^+^ Treg was significantly increased at T5 (Figure 3D). We also noticed a significant increase in the concentration of CTLA-4^+^ Treg at T4 and T5 (Figure 3E).

### Surgery-induced M-MDSCs expand Treg *in vitro*

**Figure 4:**
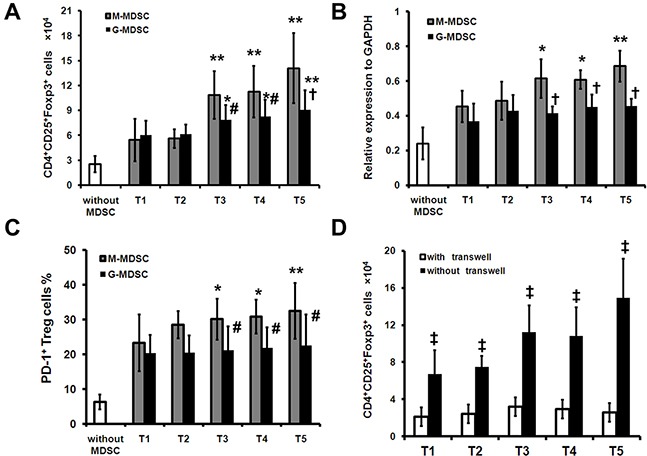
Surgery-induced M-MDSCs from lung cancer patients mediate expansion of Treg *in vitro* CD4^+^ cells isolated preoperatively from PBMCs of lung cancer patients (n=16) were cocultured with purified pre- or postoperative autologous M-MDSCs or G-MDSCs in the presence of mitomycin C-treated allogeneic PBMCs stimulator cells for 5 days. Treg were analyzed with FCM. **A**. Results are shown for combined results for four independent experiments showing absolute number of Treg. **B**. The expression of Foxp3 mRNA was analyzed by quantitative real-time PCR. **C**. The percentage of PD-1^+^ cells in Treg was analyzed by FCM. **D**. M-MDSCs were cocultured with CD4^+^ cells as described and transwell inserts were used as indicated. Shown are cumulative results from 3 independent experiments. * *P* < 0.05 and ** *P* < 0.01 as compared with T1; # *P* < 0.05 and † *P* < 0.01 as compared with “M-MDSC”; ‡ *P* < 0.01 as compared with “with transwell”.

Since the results suggest that M-MDSCs may participate in the expansion of Treg, we next investigated whether the surgery-induced M-MDSCs were capable of inducing Treg in autologous CD4^+^ T cells. We found that in the presence of M-MDSCs sorted from T3, T4 and T5, the number of Treg cells was significantly increased as compared with G-MDSCs (Figure 4A). Significant increases of Foxp3 and PD-1 expression were also detected when the M-MDSCs sorted from T3, T4 and T5 were cocultured with CD4^+^ T cells as compared with G-MDSCs (Figure 4B, 4C). Finally, the induction of Treg was cell-cell contact dependent and was abrogated when M-MDSCs and CD4^+^ T cells were separated in a transwell experiment (Figure 4D), in keeping with the pattern of Treg induction depicted previously [10].

### Surgery-induced M-MDSCs expand Treg in a mouse model

**Figure 5:**
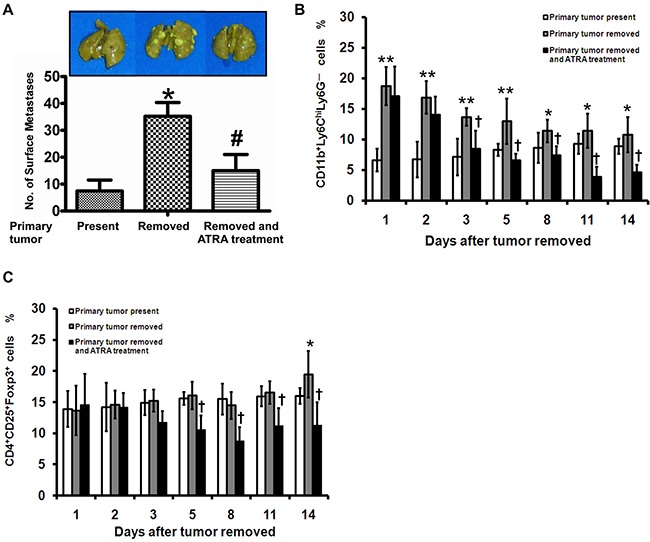
Surgery-induced M-MDSCs expand Treg and promote tumor metastasis in a mouse tumor model LLC cells were injected s.c. into the dorsa of mice. When tumors were 1,500 mm^3^ in size, the mice were divided into 3 groups and treated as described in the MATERIALS AND METHODS. **A**. All mice were killed 14 days after surgical operation, and the numbers of lung metastases on the lung surface were counted manually. Frequency of CD11b^+^Ly6C^hi^Ly6G^–^ **B**. and CD4^+^CD25^+^Foxp3^+^ **C**. cells in the PMBCs of tumor-bearing mice was analyzed. * *P* < 0.05 and ** *P* < 0.01 as compared with “primary tumor present”; † *P* < 0.01 as compared with “primary tumor removed”.

A spontaneous lung metastasis experiment was performed to examine whether tumor metastasis was promoted by an increase of surgery-induced M-MDSCs. The average metastatic nodules on the lung surface of primary tumor present and removed mice were 7.45 ± 4.08 and 35.24 ± 5.11 respectively (*P* < 0.01) (Figure 5A). In mice, MDSCs are comprised of CD11b^+^Ly6C^hi^Ly6G^–^ monocytes (M-MDSCs) and CD11b^+^Ly6C^lo^Ly6G^+^ granulocytes (G-MDSCs). Based on these cell surface markers, we found that the frequency of M-MDSCs significantly increased in tumor-bearing mice after tumor resection (Figure 5B). We also noticed a significant increase in the percentage of CD25^+^Foxp3^+^ cells in CD4^+^ T cells after tumor resection (Figure 5C). Moreover, the frequency of M-MDSCs correlated with Treg on postoperative day 8 and 14 (Supplementary Figure 1A). No correlation was found between G-MDSCs and Treg (data not shown).

There has been reported that ATRA is a potential inhibitor during induction and proliferation of MDSCs [11]. Therefore, the spontaneous lung metastasis experiment was also performed to determine the immune regulatory and anti-metastasis effects of ATRA during perioperative period. The number of metastatic nodules was 15.24 ± 6.11 in the mice treated with ATRA, which was significantly less than primary tumor-removed mice (*P* < 0.05) (Figure 5A). The percentage of the M-MDSCs in PBMCs from ATRA-treated mice decreased persistently from Day 3 after tumor resection compared with primary tumor-removed mice (Figure 5B). A significant decrease of Treg was further observed in the ATRA-treated mice compared with tumor-removed mice science Day 5 after tumor resection (Figure 5C). There was a linear association between M-MDSCs and Treg in ATRA-treated mice on postoperative day 8 and 14 (Supplementary Figure 1B). These results suggest that inhibition of M-MDSCs after surgery in tumor-bearing mice prevent Treg generation and tumor metastasis.

### Clinical outcomes

**Figure 6:**
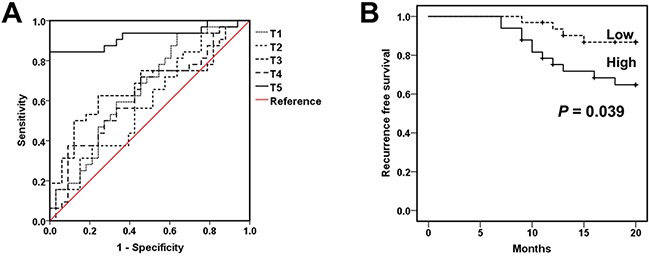
Surgery-induced M-MDSCs as a prognostic marker for lung cancer patients **A**. ROC analysis was performed in order to assess the prognostic value of M-MDSCs for lung cancer patients underwent surgical. **B**. In lung cancer patients who underwent tumor resection, the recurrence-free survival rate after treatment was compared between patients with T5-M-MDSCs^high^ (M-MDSCs > 0.435 × 10^9^/L, n = 31) and T5-M-MDSCs^low^ (M-MDSCs < 0.435 × 10^9^/L, n = 47) using the Kaplan-Meier method, with the log-rank test for comparison. T5-M-MDSCs^high^ and T5-M-MDSCs^low^, see Table 1. T5-M-MDSCs, the concentration of M-MDSCs at T5.

In order to evaluate the clinical significance of surgery-induced M-MDSCs and Treg, we assessed the viability of lung cancer patients by computed tomography (CT) or magnetic resonance imaging (MRI) scanning every 3 to 6 months after tumor resection. Receiver operating characteristic (ROC) analyses revealed that the concentration of M-MDSCs at T5 (T5-M-MDSCs) were superior to M-MDSCs levels at T1-4 as a prognostic marker for lung cancer patients underwent surgical resection (Figure 6A and Table 1). Based on the cutoff value determined in ROC analyses at T5, lung cancer patients were divided into T5-M-MDSCs^high^ and T5-M-MDSCs^low^ groups. Patients in the T5-M-MDSCs^high^ group showed significantly shorter recurrence-free survival rates than those in the T5-M-MDSCs^low^ group (*P* = 0.039) (Figure 6B), suggesting that the assessment of T5-M-MDSCs in the blood holds prognostic value. Likewise, the concentration of Treg at T5 also possessed more accuracy to predict recurrence in lung cancer patients (Supplementary Figure 2 and Supplementary Table 1).

**Table 1:** Assessment of prognostic value of M-MDSCs by ROC analyses

Parameter	Area under the curve	Sensitivity	Specificity	Cutoff value (× 10^9^/L)
T1	0.655	93.8	36.4	0.056
T2	0.591	37.5	78.8	0.249
T3	0.674	62.5	75.8	0.669
T4	0.632	37.5	90.9	0.688
T5	0.921	84.4	100	0.435

The optimal cutoff point was determined as those yielding the maximum value for (Sensitivity+Specificity-1).

## DISCUSSION

Recently, the ability of MDSCs to promote the *de novo* development of Treg cells *in vivo* has been described. In liver cancer patients, the CD14^+^HLA-DR^–^/^low^ MDSCs were significantly increased and the MDSCs exert their immunosuppressive function through the induction of Treg [10]. Here we extended the finding in lung cancer patients, and demonstrated that M-MDSCs were significantly increased after tumor resection. Furthermore, surgery-induced M-MDSCs associated with the increase of Treg. The surgery-induced M-MDSCs in peripheral blood may serve as a better prognostic marker for lung cancer. These results suggest that certain surgery-derived factors stimulate the differentiation of M-MDSC, whose abundance correlates with the degree of immunosuppression and prognosis of lung cancer underwent tumor resection. MDSCs express high levels of both arginase and inducible nitric oxide synthase (iNOS), and a direct role for both of these enzymes in the inhibition of T cell function and induction of Treg is well established [12, 13]. Our data suggest that surgery-induced M-MDSCs have increased arginase activity and directly suppress the proliferation of CD4^+^ T cells *in vitro*. In addition, transwell experiments showed that additional factors possibly acting through direct cell–cell contact play a crucial role in generation of Treg. It will be important to determine which factors are responsible for induction of Treg by surgery-induced M-MDSCs. Identification of more specific markers for surgery-induced M-MDSCs as well as Treg will help in the future to understand the biology of these 2 cell types with immunosuppressive functions.

Following a major surgery, cell-mediated immunity remains suppressed for several days with decrease in circulating levels of cytotoxic T lymphocytes (CTLs), natural killer (NK) cells, dendritic cells (DCs) and T-helper cells [14, 15]. The magnitude of this immune suppression is proportional to the degree of surgical manipulation [16]. Likewise, as we showed in the present study, the peripheral M-MDSCs increased 24 hours after surgery and lasted 1-2 weeks in both lung cancer patients and tumor-bearing mouse. We further clarified a positive correlation between the peripheral M-MDSCs and the numbers of lung metastasis induced after surgery. Therefore, the immediate postoperative immune suppresion may contribute substantially to the risk of subsequent emergence of metastasis, and perioperative modulations that reduce this risk may improve long-term prognosis. In addition to cell-mediated immunity, fully disclosing the mechanisms responsible for mediating the effects of surgical stress on tumor mass is crucial for determining the full effect of surgical manipulation on tumor metastasis and for devising effective interventions.

There is increasing evidence from preclinical and clinical studies that MDSCs play an important role in all steps leading to metastasis [17]. Although the immune suppressive activity of MDSCs is extremely important for the formation of a metastatic niche [18, 19], these cells employ a number of other mechanisms promoting metastases. An increase in IDO-expressing MDSCs in breast cancer correlated with increased lymph node metastasis in breast cancer patients [20]. In patients with melanoma, the development of metastases and poor survival was associated with increases in both circulating CD11b^+^CD14^−^CD15^+^ polymorphonuclear-MDSCs and M-MDSCs. These changes are associated with changes in plasma and cellular levels of immune regulatory microRNAs [21, 22]. In this report, we demonstrate that surgery-induced M-MDSC are able to expand Treg, and are associated with increased tumor metastases, suggesting a possible mechanism for the emergence of metastases following tumor resection. One limitation of the present study is that this is a small patient cohort with relatively short postoperation follow-up. Moreover, the role of each of these cell populations and their exact mechanism of action in postoperative tumor metastasis remains to be defined.

In summary, we found that surgery-induced M-MDSCs were significantly increased and were more efficient in inhibiting the proliferation of T cells and mediating the development of Treg. Furthermore, the accumulation of surgery-induced M-MDSCs and Treg correlated with postoperative tumor metastasis, thus holding prognostic value. Thus, we propose a possible mechanism by which surgery induces immune-suppressive ingredients and promotes tumor metastasis.

## MATERIALS AND METHODS

### Patients

A total of 111 newly diagnosed adult lung cancer patients were prospectively enrolled at the First Affiliated Hospital of Kunming Medical University between July 2014 and December 2015. Ethics Committee approval was obtained from the Internal Review Board of Kunming Medical University and a written informed consent was obtained in accordance with the declaration of Helsinki. Peripheral blood was drawn at preoperation, intraoperation and on postoperative day 1, 3 and 7. Analyzing the peripheral blood by using an automated hematology analyzer, the concentration of PBMCs was obtained.

### Tumor cell line and mice

The lewis lung carcinoma (LLC, H-2^b^) cell line was obtained from American Type Culture Collection (ATCC; Rockville, MD, USA) and maintained in RPMI 1640 medium (Gibco-BRL, Carlsbad, CA) supplemented with 10% fetal calf serum, 30 μg/mL gentamicin, and 0.2% sodium bicarbonate. Inbred female C57BL/6 (B6, H-2^b^) (8–10 weeks) mice were purchased from the Experimental Animal Institute of Peking Union Medical College.

### Flow cytometry analysis and cell sorting

To determine the frequency and phenotype of MDSCs and Treg in PBMCs from lung cancer patients, flow cytometry (FCM) analysis was done using the following fluorescein-conjugated mouse anti-human monoclonal antibodies (mAb): CD4, CD11b, CD14, CD25, CD33, Foxp3, HLA-DR, PD-1 and CTLA-4 (BD Pharmingen, San Diego, CA). Peripheral blood was collected from B6 mice through tail vein and stained with fluorescein-conjugated rat anti-mouse CD4, CD11b, CD25, Foxp3, Ly6C and Ly6G mAb (BD Pharmingen). FCM analysis of M-MDSCs, G-MDSCs and Treg in lung cancer patients was performed according to the gating protocol showed in Supplementary Figure 3 on Beckman Coulter FC500 (San Jose, CA) and FCM data was analyzed using CXP software (Beckman Coulter). The concentration of M-MDSCs, G-MDSCs and Treg in lung cancer patients was calculated according the formula: = % of M-MDSCs, G-MDSCs or Treg in PBMCs × concentration of PBMCs.

Human PBMCs were isolated from freshly heparinized peripheral blood from lung cancer patients by standard Ficoll density gradient centrifugation (GE Healthcare, Pittsburgh, PA). Isolation of M-MDSCs, G-MDSCs and Treg was performed according to the gating protocol showed in Supplementary Figure 3 on FACSVantage SE cell sorter (Becton Dickinson, Franklin Lakes, NJ).

### MDSCs suppression assay

CD3^+^ cells were isolated from PBMCs using anti-CD3 magnetic beads (Miltenyi Biotec, Bergisch Gladbach, Germany) and plated at 2×10^5^ cells/well in 1 μg/ml of anti-CD3 mAb (muromonabCD3, Janssen Pharmaceutica, Titusville, NJ)-coated plates. Isolated 1×10^5^ M-MDSCs from the same lung cancer patient were added to the wells. CD3^+^ cell proliferation was determined 72 hours later after incubating with ^3^H-thymidine for the last 16 hours. ^3^H-thymidine uptake was counted using a liquid scintillation counter and expressed as counts per minute (cpm). The percentage of T cell proliferation was calculated according to the formula: T cell proliferation (%) = (the cpm in M-MDSCs and CD3^+^ cells coculture) / (the cpm in CD3^+^ cell alone) × 100%.

### Reverse transcription-PCR (RT-PCR) and quantitative real-time PCR

M-MDSCs and G-MDSCs were isolated and total RNA was extracted using TaKaRa RNAiso Reagent (Takara Bio Inc. Otsu, Japan) according to the manufacturer’s instructions. A RT-PCR procedure was used to determine relative quantities of mRNA (One-step RT-PCR kit, Qiagen). The primers for all genes tested, including internal control GAPDH, were synthesized by Invitrogen: GAPDH 5′ - AGCCACATCGCTCAGACAC -3′ (sense) and 5′ - GCCCAATACGACCAAATCC -3′ (antisense), Foxp3 5′ – CTACGCCACGCTCATCCGCTGG -3′ (sense) and 5′ – TAGGGTTGGAACACCTGCTGGG -3′ (antisense). For quantitative real-time PCR, cDNA (2 μL) reverse transcribed from total RNA was amplified by real-time PCR with 1 SYBR Green Universal PCR Mastermix (Bio-Rad). Each sample was analyzed in duplicate with the IQ-Cycler (Bio-Rad) and the normalized signal level was calculated based on the ratio to the respective GAPDH housekeeping signal.

### Determination of arginase activity

The arginase activity of M-MDSCs was determined as described before [5]. A standard curve consisting of serial dilutions of urea was run in parallel. The urea concentration was measured at 540 nm.

### Treg generation *in vitro*

CD4^+^ T cells were isolated preoperatively from PBMCs obtained as baseline from lung cancer patients using anti-CD4 magnetic beads (Miltenyi Biotec). CD4^+^ T cells (2 × 10^5^) were then cultured with M-MDSCs or G-MDSCs (4 × 10^4^) from lung cancer patients in a ratio of 5:1 in the presence of stimulator cells. CD4-depleted PBMCs (4 × 10^4^) from healthy donor were incubated with mitomycin C for 30 min and used as stimulator cells. After 5 days, T cells were recovered and stained with CD4, CD25 and Foxp3.

### Experimental tumor metastasis model

LLC spontaneous pulmonary metastasis experiments were performed as described previously [23]. Briefly, 1 × 10^6^ LLC cells in 0.1 mL of PBS were injected s.c. into the dorsa of mice. When tumors were 1,500 mm^3^ in size, 14 days after LLC inoculation, the mice underwent surgical removal of the tumor. The incision was closed with simple interrupted sutures. After tumors were removed, the mice were randomized and treated with ATRA (Sigma-Aldrich, St. Louis, MO) or PBS. On Day 28 after LLC inoculation, all mice were killed and the numbers of lung metastases on the lung surface were counted manually with macroscopic examination.

### Statistical analysis

Results were presented as mean ± standard deviation (SD). Student’s *t*-test was used to compare means between 2 groups. Multiple comparisons between more than two groups were analyzed by analysis of variance (ANOVA) or Kruskal-Wallis nonparametric test. The correlation between two groups was assessed by Pearson’s analysis. The recurrence-free survival rate in patients with lung cancer who underwent surgical resection was compared using the Kaplan-Meier method with the log-rank test for comparison. Differences were considered significant when *P* < 0.05. Statistical analysis was performed using commercially available software (PASW Statistics 18).

## SUPPLEMENTARY FIGURES AND TABLE


