# Supplementary material for: Surgery-induced monocytic myeloid-derived suppressor cells expand regulatory T cells in lung cancer
Source: Oncotarget. 2017 Feb 4;8(10):17050–8. doi: 10.18632/oncotarget.14991 (PMC5370021; doi:10.18632/oncotarget.14991)
Supplement: Supplementary file 1 [file oncotarget-08-17050-s001.pdf]

Surgery-induced monocytic myeloid-derived suppressor cells expand regulatory T cells in lung cancer

SUPPLEMENTARY FIGURES AND TABLE

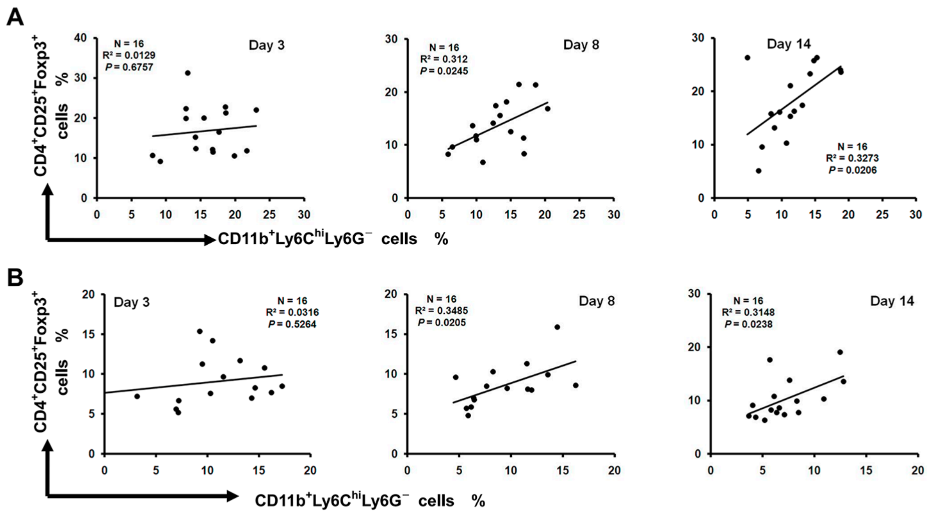

Supplementary Figure 1: Correlation between the percentage of M-MDSCs and Treg in tumor-bearing mice. Linear association between the percentage of M-MDSCs and Treg in primary tumor removed **A.** and ATRA-treated mice **B.** on postoperative day 3, 8 and 14.

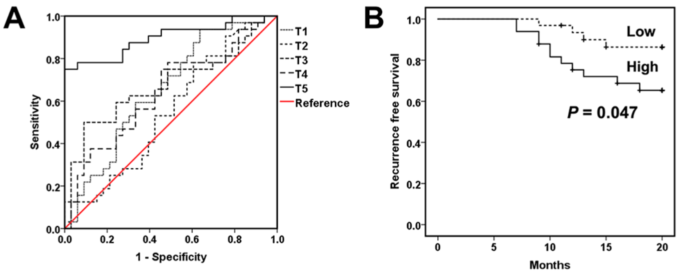

Supplementary Figure 2: Clinical significance of surgery-induced Treg in lung cancer patients. A. ROC analysis was performed in order to assess the prognostic value of Treg for lung cancer patients underwent surgical. **B.** Recurrence-free survival rate was compared between patients with T5-Treg^{high} (Treg > 0.032 × 10^9/L, n = 30) and Treg^{low} (Treg < 0.032 × 10^9/L, n = 48). T5-Treg^{high} and T5-Treg^{low}, see Supplementary Table S1. T5-Treg, the concentration of Treg at T5.

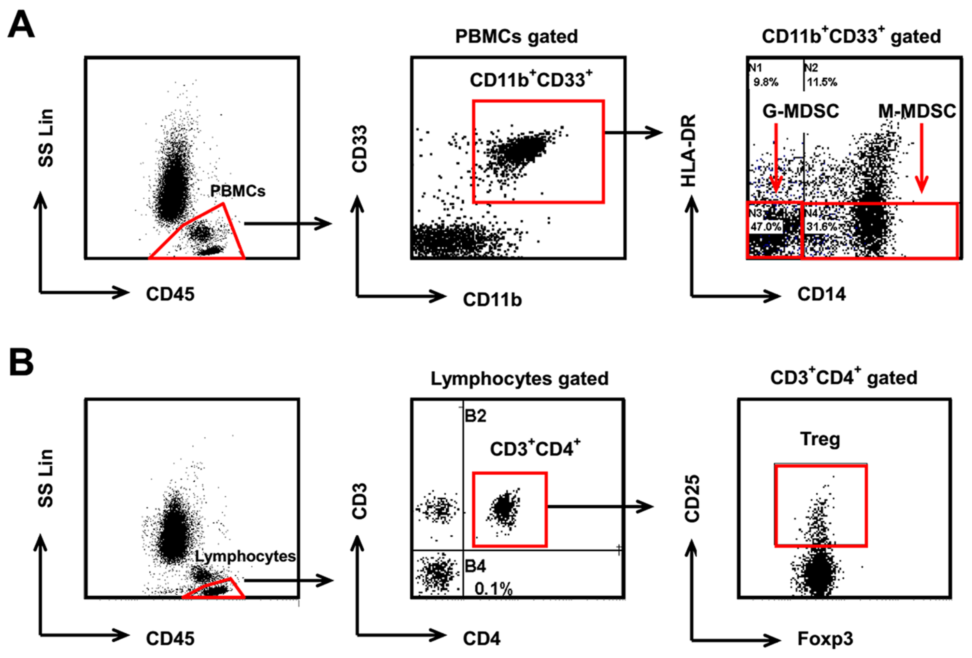

Supplementary Figure 3: Phenotypic analyses of M-MDSCs, G-MDSCs and Treg in peripheral blood. Gating protocols and representative dot plots of M-MDSCs, G-MDSCs **A.** and Treg **B.** in a lung cancer patient were shown.

Supplementary Table 1: Assessment of prognostic value of Treg by ROC analyses

Parameter	Area under the curve	Sensitivity	Specificity	Cutoff value($\times 10^9$/L)
T1	0.652	93.8	36.4	0.023
T2	0.554	78.1	39.4	0.014
T3	0.674	50.0	90.9	0.021
T4	0.642	78.1	51.5	0.026
T5	0.897	75.0	100	0.032

The optimal cutoff point was determined as those yielding the maximum value for (Sensitivity+Specificity-1).
